# Premotor and Motor Cortices Encode Reward

**DOI:** 10.1371/journal.pone.0160851

**Published:** 2016-08-26

**Authors:** Pavan Ramkumar, Brian Dekleva, Sam Cooler, Lee Miller, Konrad Kording

**Affiliations:** 1 Sensorimotor Performance Program, Rehabilitation Institute of Chicago, Illinois, 60611, United States of America; 2 Department of Physical Medicine and Rehabilitation, Northwestern University, Chicago, United States of America; 3 Department of Biomedical Engineering, Northwestern University, Chicago, United States of America; 4 Department of Physiology, Feinberg School of Medicine, Northwestern University, Chicago, United States of America; 5 Northwestern University Interdepartmental Neuroscience Program, Chicago, United States of America; SUNY Downstate MC, UNITED STATES

## Abstract

Rewards associated with actions are critical for motivation and learning about the consequences of one’s actions on the world. The motor cortices are involved in planning and executing movements, but it is unclear whether they encode reward over and above limb kinematics and dynamics. Here, we report a categorical reward signal in dorsal premotor (PMd) and primary motor (M1) neurons that corresponds to an increase in firing rates when a trial was not rewarded regardless of whether or not a reward was expected. We show that this signal is unrelated to error magnitude, reward prediction error, or other task confounds such as reward consumption, return reach plan, or kinematic differences across rewarded and unrewarded trials. The availability of reward information in motor cortex is crucial for theories of reward-based learning and motivational influences on actions.

## Introduction

How the brain learns based on action outcomes is a central question in neuroscience. Theories of motor learning have usually focused on rapid, error-based learning mediated by the cerebellum, and slower, reward-based learning mediated by the basal ganglia (for a review, see [1]). Different combinations of reward and sensory feedback result in different learning rates. For instance, positive and negative rewards influence motor learning differently [2, 3]. When reward is combined with sensory feedback, it can accelerate motor learning [4]. Reward is thus a fundamental aspect of learning [5, 6, 7, 8]. Various reward signals have been characterized in the midbrain, prefrontal and limbic cortices [9, 10, 11, 12, 13]. Yet, we do not know how neurons in the motor system obtain the reward information that could be useful for planning subsequent movements.

The dorsal premotor cortex (PMd) and the primary motor cortex (M1) are known to be involved in planning and executing movements. We know this because movement goals (e.g., direction of upcoming movement), kinematics (e.g., position, velocity and acceleration) and dynamics (e.g. forces, torques, and muscle activity) are reflected in the firing rates of motor cortical neurons [14, 15, 16, 17, 18, 19, 20]. If movement plans need to be modified based on previous actions, then information about their outcomes must reach motor cortices. In many real world settings, task outcomes typically manifest in the form of reward.

Recently, Marsh et al. [21] have shown a robust modulation of M1 activity by reward expectation both during movement and observation of movement. To further investigate the nature of this potential reward signal, we trained monkeys to reach to targets based on noisy spatial cues and rewarded them for correct reaches. We induced different reward expectation on a trial-by-trial basis and quantified the representation of reward in PMd and M1. We observed that ~28% of PMd neurons and ~12% of M1 neurons significantly modulated their firing rates following trials that were not rewarded. The effect could not be explained simply by kinematic variables such as velocity or acceleration, reward consumption behavior, or upcoming movement plans, nor by task variables that may bias successful task performance, such as the noise in the target cue, the reward history, or the precision of the reach. This effect might constitute an important piece in the larger puzzle of how motor plans are modified based on reward.

## Results

**Fig 1:**
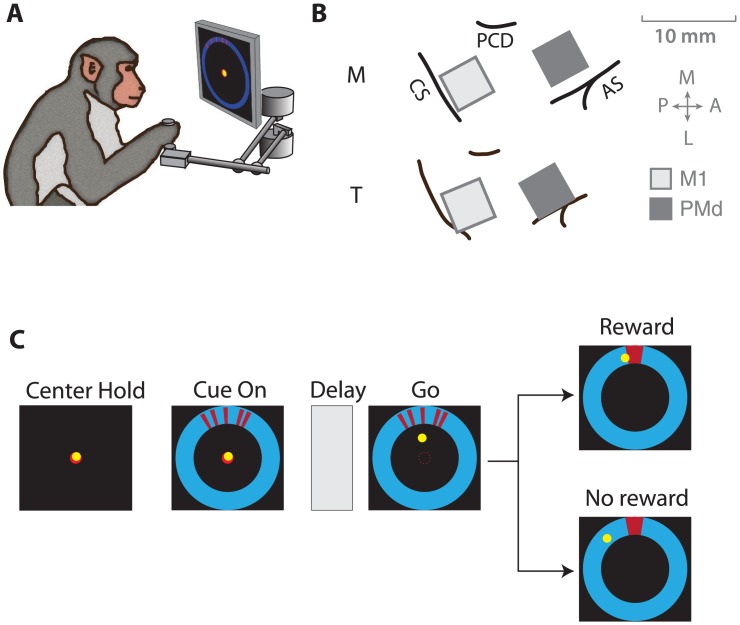
Reaching task to uncertain targets. **(A)** Monkeys made center–out reaches using a planar manipulandum that controlled an on-screen cursor. (**B**) We recorded from chronic microelectrode arrays implanted in dorsal premotor cortex (PMd) and primary motor cortex (M1). CS: central sulcus, AS: arcuate sulcus, PCD: precentral dimple (**C**) Monkeys reached towards a target that was cued using a set of 5 line segments, whose dispersion varied from trial to trial. In each trial, the true target location was sampled from a von Mises “prior” distribution centered at 90° (clockwise from the rightmost point on the annulus) with one of two concentration parameters specifying a broad or narrow prior. The line segments making up the cue were then sampled from a “likelihood” von Mises distribution centered on the target location, with one of two concentration parameters (κ = 5 or 50 for Monkey M and κ = 1 or 100 for Monkey T) specifying a broad or narrow spread. Adapted from Dekleva et al., 2016.

Our goal in this study was to investigate whether the motor system—in addition to planning and executing actions—also encodes responses to reward, which are key for learning about the environment and modifying motor plans. To this end, we trained two macaque monkeys to make center–out reaches to uncertain targets and rewarded them for successful reaches. The monkeys made reaching movements while grasping the handle of a planar manipulandum, their hand position represented by an on-screen cursor (Fig 1A). During this task, we recorded from two 96-channel microelectrode arrays (Fig 1B, Blackrock Microsystems), chronically implanted in the primary motor cortex (M1) and the dorsal premotor cortex (PMd).

A trial began when the monkey moved the cursor to the central target (Fig 1C). The true location of the target was not shown. Instead, a noisy cue was presented at a 7-cm radial distance from the center to indicate the approximate location of the outer target. The cue comprised a cluster of line segments generated from a distribution centered on the true target. Monkeys were trained to reach only after a combined visual/auditory go cue, which was delivered after a variable (0.8–1.0 s) delay period following cue appearance. At the end of the reach, the actual circular target (15° diameter) was displayed. If the monkey had successfully reached the target, an appetitive auditory cue announced the subsequent delivery of a juice reward. If the reach ended outside the target, an aversive auditory cue announced the failure of the reach and no reward was delivered. After the end of the trial, the monkey was cued to return to the center target in order to begin the next trial. The median inter-trial interval across animals and sessions was 2.79 ± 0.25 seconds. On any given trial, since the actual target was not shown, the monkey had to infer its location, potentially by combining the information in the noisy target cue with prior knowledge accumulated about the target location in previous trials (for details, see [22]).

We assume that the monkeys calibrated their expected reward based on the cue uncertainty. To manipulate their reward expectation, we varied cue uncertainty from trial to trial. Specifically, we determined the dispersion of the line segment cluster on each trial by drawing the location of each line segment from either a narrow or a broad distribution (see Fig 1 for details). A narrower spread of line segments indicated the target location with lesser uncertainty than a broader spread. To verify that animals indeed change their reward expectation, we looked at the latency of movement onset after the go cue. We found that animals indeed started their reach later on average when they were more uncertain about the target location (35 ± 28 ms for narrow spreads; 111 ± 16 ms for broad spreads; mean ± SEM across animals and sessions). Thus, manipulating the dispersion on each trial is likely to have induced trial-by-trial changes in reward expectation.

### Neural coding of reward

**Fig 2:**
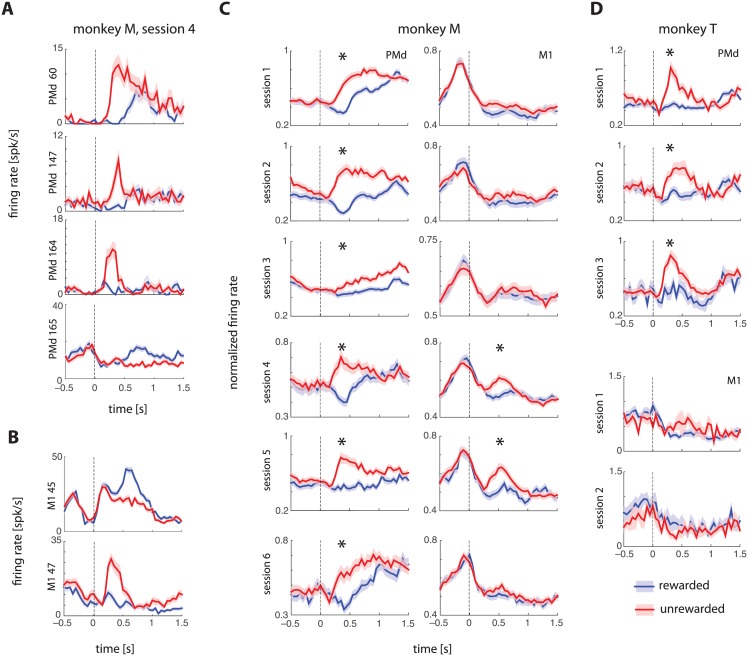
Neural coding of reward. **(A, B)** Example neurons from PMd and M1 showing reward modulation that persisted after controlling for kinematic differences between rewarded and unrewarded trials (see Fig A in S1 File). Error bars show standard errors (SEMs) across trials. Vertical dashed line at zero indicates the time of reward. **(C, D)** Trial-averaged, population-averaged normalized firing rates (mean ± SEMs across neurons) for PMd and M1 from two monkeys. For each session, significant differences between rewarded and unrewarded peak PSTH amplitudes are indicated using an asterisk. PMd shows a clear increase after unrewarded trials compared to kinematically-matched rewarded trials.

We asked if the firing rates of PMd and M1 neurons indicated whether a reward was obtained in the trial by comparing the peristimulus time histograms (PSTHs) aligned to the end of the trial timestamp (corresponding to the auditory cue that indicated whether a reward will be delivered) for rewarded and unrewarded trials. We matched the kinematics of the trials across the two conditions to control for trivial firing rate consequences of behavioral differences (see Fig A and Table A in S1 File for details about matching kinematics). We found that ~28% of PMd neurons and ~12% of M1 neurons modulated their firing rates in response to reward or lack thereof. Nearly 25% of all PMd neurons recorded increased their firing rates following unrewarded trials compared with rewarded trials (Fig 2A and 2B show example PMd and M1 neurons from one session). In comparison, only ~3% of PMd neurons increased their firing rates after rewarded trials. For M1, these numbers were ~8% and ~4%, respectively.

We then determined the extent to which this effect was visible across the entire population. To do so, we normalized single neuron PSTHs computed from kinematically-matched trials by setting the peak of each PSTH to 1, and computing separate PTSHs for M1 and PMd. There was a significant increase in population-wide normalized firing rate following unrewarded trials in both monkeys (Fig 2C and 2D). PMd had a significant effect in all 9 sessions (6 from Monkey M and 3 from Monkey T), whereas the effect in M1 was significant in only 2 out of 9 sessions.

The firing rate effect of unrewarded trials in PMd and to a lesser extent, M1, is completely confounded by the fact that only successful trials were rewarded. Thus, increased firing rate for unrewarded trials could potentially be an intrinsic signal of success or failure, or might indicate some other correlate of the outcome of a goal-directed movement. To eliminate this confound, we ran a separate experiment on one monkey (Monkey M) in which we withheld reward in a subset of successful reaches. We found that firing rates increased even for these successful but unrewarded trials (Fig B in S1 File), suggesting that the increased activity following unsuccessful trials is related to lack of extrinsic reward, not an intrinsic measure of task outcome.

### Putative reward signal is not explained away by task confounds

Several other variables could potentially confound this putative reward signal as well. As we did for kinematic differences (Fig A in S1 File), examining groups of rewarded and unrewarded trials that are matched for these confounding variables is a potential means of disambiguating the source of the effect. Yet, adequately matching all possible confounding variables is impossible because of the trade-off between precise matching of numerous potential confounds, and adequate remaining sample size. Instead, we controlled for potential confounds by using multiple linear regression models of trial-by-trial firing rates. Specifically, we modeled single neuron spike trains using Poisson generalized linear models (GLMs) (see e.g., [23, 24, 25], and Methods for details).

To construct the GLM, we modeled neural spike counts during a 2-second epoch (–0.5 to 1.5 seconds, in 10-ms bins) around the reward onset. Spike counts were modeled as a function of the reward, which we represented as a binary variable (+1 for rewarded trials, –1 for unrewarded trials), aligned to the reward onset. In addition, we included the following confounding variables in the multiple regression.

1. *Kinematics*. PMd and M1 neurons are known to encode kinematic variables during movement planning and execution [16, 17, 26]. Therefore, we included instantaneous velocity and acceleration time series, binned in 10-ms time bins.
2. *Uncertainty*. Previous work has suggested that PMd can encode plans for more than one potential target [27] and we have recently shown that PMd encodes uncertainty about the reach target location [22]. Further, cue uncertainty influences the likelihood of a successful outcome, since monkeys are more successful in low-uncertainty trials. Although we did not find an effect of uncertainty on PSTHs aligned to reward time (Fig C in S1 File), we included a measure of trial-specific uncertainty as a confounding variable. Specifically, we used the dispersion of the target cue line segments, where dispersion is the largest circular distance between all possible pairs of line segments.
3. *Reward history*. The outcome of the previous trial (and more generally, the history of reward) can influence the level of satiety, and thus the motivation and perceived value of a potential reward [12]. To control for this possibility, we included the previous trial’s outcome as a binary covariate (+1 for success, –1 for failure).
4. *Error*. The reward-related signal might be useful for reinforcement learning (temporal difference learning) if it encoded some information about the discrepancy between the reach direction and the true target direction (presented visually at the end of the trial). To test whether PMd/M1 neurons encode error magnitude, we included the unsigned reaching error (reach precision) as a covariate.
5. *Return goal*. Another potential confound is that the movement plan for the return reach to the center target may be modulated by recently obtained reward. Although a separate control analysis (Fig D in S1 File) suggested there were no systematic differences between return reach planning for rewarded and unrewarded trials, here we controlled for this possibility by including a covariate that specified the return reach direction; in particular, we used two covariates specifying the direction cosines (cosine and sine) of the return reach direction.

Environmental covariates—uncertainty, error, and reward—that are potential causes of firing rate changes, invariably *lead* spikes in M1 and PMd. By contrast, movements are the consequence of motor cortical activity. Therefore, kinematic variables are likely to *lag* spikes. To model these latency differences for the different covariates, we used temporal basis functions (see Methods for details).

**Fig 3:**
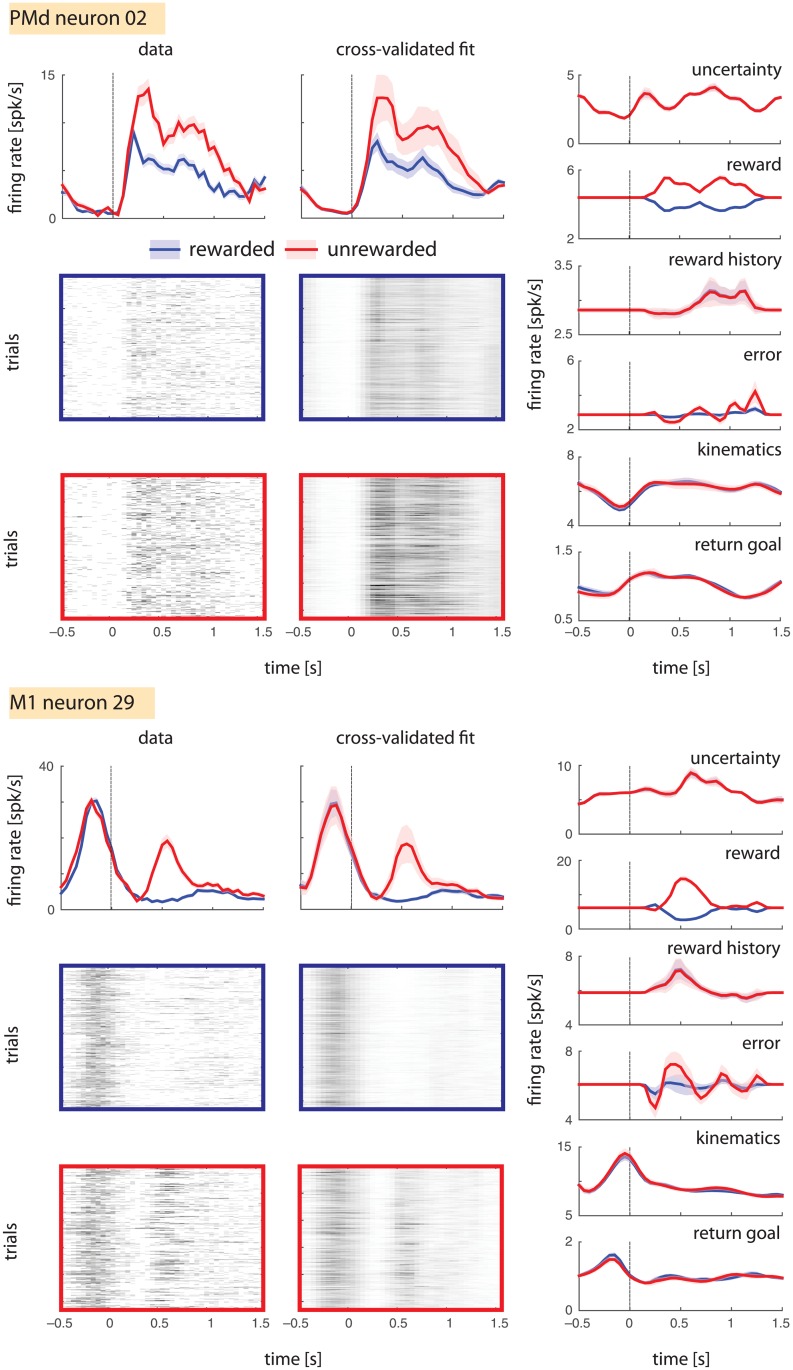
Generalized linear modeling of reward coding. Model predictions for two example neurons are shown. Left: PSTHs for rewarded (blue) and unrewarded (red) trial subsets are shown for the test set, along with corresponding single-trial rasters for both data and model predictions on the test set. Right: Component predictions corresponding to each covariate.

Our model accurately captured the reward-related activity of many neurons. Comparing the data and cross-validated fit (see Methods) panels of Fig 3, we see that the trial-averaged data PSTHs and trial-averaged model-predicted firing rates are extremely similar. Across 70 PMd and 191 M1 neurons in a representative session (Monkey M, session 4), the model explained almost all the variance in the trial-averaged data (mean ± standard deviation of *R*^*2*^ = 0.96 ± 0.05 and 0.93 ± 0.08 for PSTHs averaged across successful and unsuccessful trials, respectively). These high *R*^*2*^s suggest that the model includes almost all potential sources of predictable variance. Therefore, if the reward covariate cannot be explained away by the confounding covariates, it is likely that the neurons represent reward.

To understand whether the reward covariate explains a significant fraction of the variance, we visualized the predictions of individual model covariates (reward, kinematics, uncertainty, reward history, error, and return goal), for rewarded and unrewarded trial subsets. Among all model covariates, only the reward covariate made different predictions for firing rates in rewarded and unrewarded trials (Fig 3, right panels); the predictions of other covariates were similar across these conditions. This preliminary analysis of example neurons seemed to suggest that reward was indeed the predominant driver of firing rate variance.

To quantify the marginal effects of reward, kinematics and other confounding variables, we also built partial models leaving each covariate out and then comparing these respective partial models against the full model using the relative pseudo-*R*^*2*^ metric as a measure of effect size (see Methods for definitions and details). Briefly, we used two-fold cross-validation to quantify error estimates on pseudo-*R*^*2*^s. We used each half of the data as a training set to fit the full and partial models and computed the pseudo-*R*^*2*^s on the other half (the test set). We obtained 95% confidence intervals (CIs) on the cross-validated test set pseudo-*R*^*2*^ by bootstrapping on the test sets and used these to determine which neurons were significantly predicted by the covariate of interest at the 2σ and 5σ significance levels (see Methods).

**Fig 4:**
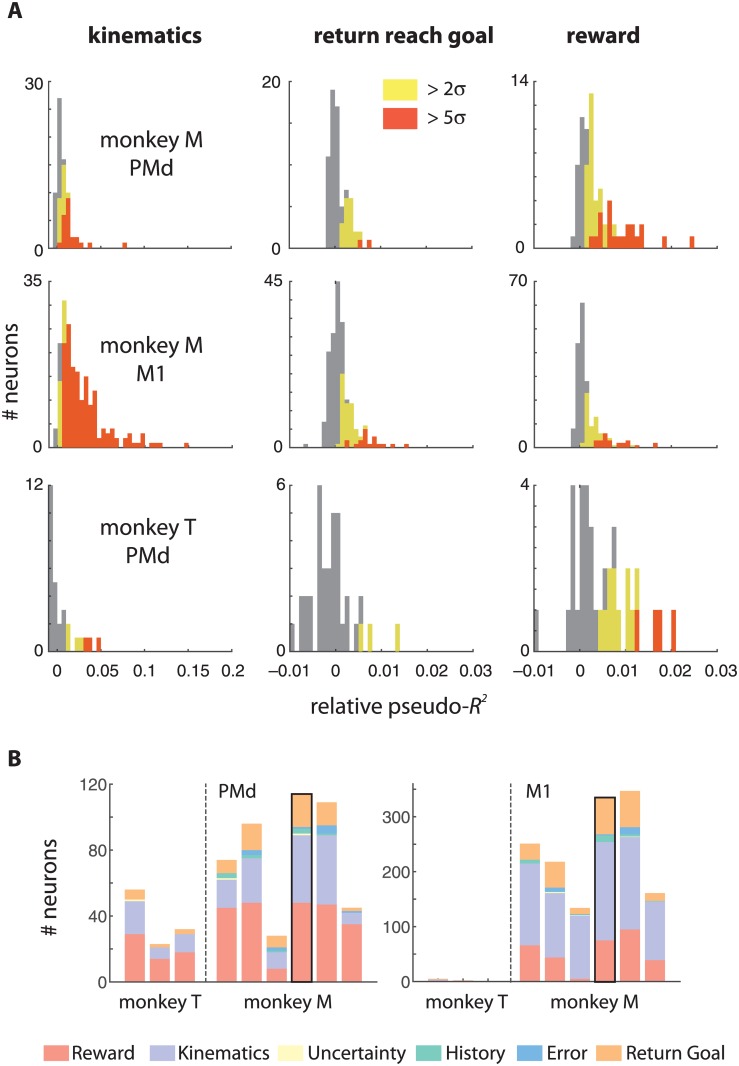
Across-session summary of reward encoding. (**A**) The distribution of effect sizes across the population from one session in each monkey (Monkey M, Session 4 and Monkey T, Session 1) as measured by mean relative pseudo-*R*^*2*^s are shown for kinematics, return reach goal, and reward. A large fraction of neurons in both PMd and M1 remained statistically significantly modulated by reward after controlling for cue uncertainty, reward in the previous trial, error magnitude, instantaneous kinematics, and planning of the return reach. (**B**) A stacked bar shows the number of significant neurons at the 2-sigma level for each session. The representative session for which histograms of effect sizes are shown above is indicated with a black border.

Three of the covariates accounted for a large fraction of the variance in PMd and M1 firing rates. As expected, a large number of neurons (41/70 in PMd, and 179/191 in M1; 2σ significance criterion; Fig 4A, left panel) were significantly modulated by reach kinematics (instantaneous velocity and acceleration) in a representative session (Monkey M, session 4). Further, many neurons (20/70 PMd and 67/191 M1; Fig 4A, middle panel) encoded the direction of the upcoming return reach. However, a large fraction of PMd (48/70), and M1 (75/191) neurons also encoded reward (Fig 4A, right panel). By comparison, a negligible number of neurons in either PMd or M1 encoded cue uncertainty (1), error magnitude (3) or reward history (15)—these were likely false positives that did not survive a multiple-comparison correction. These results were very similar across multiple sessions in both monkeys (Fig 4B). For Monkey T, the quality of the M1 array had degraded at the time of these experiments, and the spike-sorted neurons had extremely low firing rates, insufficient to fit reliable multivariate GLMs. Therefore we were only able to quantify the effects in PMd.

In the generalized linear models, we could not directly control for actual reward consumption, which involves mouth and neck movements, and might therefore affect firing rates in PMd and M1. However, we mainly observed *increases* in neural activity when reward was not received. This would only be possible if the monkey had made more vigorous mouth or neck movements in the absence of reward, than while actually consuming reward (e.g. by potentially sucking harder on the tube when no reward was delivered). We could not quantitatively control for this possibility because we did not measure kinematics or EMG signals from the face. However, we made doubly sure that the monkey correctly interpreted the auditory cue signaling lack of reward. To do this, we filmed one monkey during a separate session (Monkey M; see Fig E in S1 File) and observed that it simply sat still during trials when no reward was delivered, without making any oral contact with the reward delivery tube. Therefore, the robust reward signal cannot be explained by reward consumption. Taken together, our results suggest that a large fraction of PMd and M1 neurons encode the outcome of the task independently of uncertainty, error magnitude, kinematics, reward history, reward consumption, and the return reach plan.

## Discussion

We asked if the premotor and motor cortices, implicated in planning and executing movements, might also represent the reward associated with those movements. We found a strong representation of reward in PMd firing rates, with a lesser effect in M1. The increase in firing rates was observed in response to the absence, and to a lesser extent, the occurrence of extrinsic reward, but not the intrinsic success or failure of the trial. We then asked if the reward signal encoded motivation or satiety (modeled by reward history), prediction error (modeled by cue uncertainty), or movement precision (modeled by error magnitude), but found no evidence for any such signals. We also confirmed that although kinematics and return movement planning could explain firing rate variance, neither of them could explain away the reward signal. Although the motor cortex has traditionally been thought of as a brain area that sends control signals to the spinal cord and muscles, recent studies (such as [21]) including ours, establish the important additional effect of reward on motor cortex activity.

One weakness of this experiment is the lack of statistical power to ask if there were trial-by-trial reward dependent learning effects. The target location and the dispersion of the cue lines were drawn at random on each trial; hence, there was very little opportunity to transfer knowledge from one trial to the next. As trial-by-trial learning is generally relatively slow (and further slowed by high feedback uncertainty [28] we expected only a small trial-by-trial effect. Not surprisingly then, we did not find that the reward signal was directly tied to the behavioral performance of subsequent trials. If and how the reward signal does influence trial-by-trial learning should be investigated with further experiments. A second weakness of our design, which is typically common across many animal experiments, is that it does not rule out the possibility of covert motor rehearsal following error or lack of reward. Such rehearsal might activate premotor and motor cortices without resulting in overt behavior. Although we rule out any direction-specific effects of the reported reward outcome signal, it is impossible to definitively rule out the influence of covert rehearsal. This is an important constraint that future experiments must contend with.

Reward is a central feedback mechanism that regulates motivation, valuation, and learning [8, 9]. Existing computational theories of this phenomenon, such as reinforcement learning and temporal difference learning [5, 7], have been successful in explaining the dopaminergic prediction error signal, but reward coding in the brain is far more heterogeneous and pervasive than just that [6, 10]. The large majority of brain areas implicated in reward processing, such as basal ganglia, ventral striatum, ventral tegmental area, and the orbitofrontal cortex, are predictive in nature, with predictions including reward probability, reward expectation, and expected time of future reward (for a review see [9]). Dopaminergic neurons in the ventral striatum also encode the mismatch between predicted and obtained rewards, combining reward prediction with reward feedback. Thus far, only the lateral prefrontal cortex has been shown to encode reward feedback without any predictive component. To our knowledge, the previous studies examining reward-related signaling in the premotor and motor cortices [11, 12, 21] reported a predictive code for reward magnitude and reward expectation but not for reward outcome or feedback. Previous studies have implicated the motor cortex in error-related signaling [29, 30]. Here, we show for the first time that single neurons in premotor and motor cortices encode reward-related feedback. Our finding adds another piece to the heterogeneity of reward representation in the midbrain and cortex, which will help extend future theories of reward-based learning.

The latency of the reward signal in PMd and M1 is on the order of 400–600 milliseconds. This latency is much slower than the rapid (~100 ms) reward prediction error signal observed in dopaminergic neurons in the midbrain [31, 32]. Thus, the pathway to reward outcome representation in the motor cortex is likely to be mediated by the basal ganglia-thalamo-cortical loop. In particular, we know the striatum, which receives projections from reward-sensitive dopaminergic neurons, feeds back to the cortex through other basal ganglia structures and the thalamus [33]. The anterior cingulate cortex is also implicated in decision-making based on past actions and outcomes [34]. This is an alternate possibility for the origin of the signal that we observe in premotor and motor cortices. Thus, it is likely that the motor cortex, along with prefrontal cortex and other areas, reflects rather than generates the reward signal.

At present, the function of this reward outcome signal in the motor cortices is unclear. A recent EEG-fMRI study [35] suggests that two distinct value systems shape reward-related learning in the brain. In particular, they found that an earlier system responding preferentially to negative outcomes engaged the arousal-related and motor-preparatory brain structures, which could be useful for switching actions if needed. Therefore, the reward signal in PMd and M1 could potentially induce the cortical connectivity changes required for correcting subsequent motor plans based on mistakes. Further investigations of our finding might thus potentially reveal the mechanisms by which the brain acquires new motor skills.

Behavioral studies of motor control are at the advanced stage of describing trial-to-trial learning and generalization to novel contexts using sophisticated Bayesian decision theory and optimal control models [3, 36, 37, 38, 39]. Yet, we are only beginning to understand how different neural systems work together to achieve these behaviors. We have shown a robust reward signal in premotor and motor cortex that is not simply the result of movement kinematics or planning. Establishing a link between this reward signal and motor learning could potentially open up a new area of research within computational motor control.

## Methods

### Surgical procedures and animal welfare

We surgically implanted two chronic 96-channel microelectrode arrays in dorsal premotor cortex (PMd) and primary motor cortex (M1) of two macaque monkeys. For further surgical details including array locations on the cortex, please see Dekleva et al., (2016). All surgical and experimental procedures were fully consistent with the guide for the care and use of laboratory animals and approved by the institutional animal care and use committee of Northwestern University. Monkeys received appropriate pre- and post-operative antibiotics and analgesics.

The monkeys are pair-housed in standard size quad cages at the Feinberg School of Medicine. They receive a standard ration of Purina monkey chow biscuits twice a day. They are provided with hammocks and numerous puzzle and foraging toys in their cages. After completing a full series of recording experiments spanning six years, we placed monkey T under deep, surgical anesthesia with an intraveneous IV injection of Euthasol (25mg/kg), prior to transcardiac perfusion with saline followed by 4% formaldehyde solution. Experiments continue with monkey M.

### Single-neuron and population PSTHs

We calculated peri-stimulus time histograms (PSTHs) of firing rates (spikes/s), in 25-ms windows aligned to the reward timestamp and averaged them across trials. Error bars were computed as standard errors of mean across trials. To test whether neurons were significantly modulated by reward, we compared mean firing rate in a [0, 1.5] second interval after the reward timestamp across rewarded and unrewarded trials using a one-sided *t*-test, with a significance level of α = 0.05, Bonferroni-corrected for the number of neurons recorded in a single session. To calculate population-averaged PSTHs, we took the mean trial-averaged PSTHs, normalized them to have a peak firing-rate of 1, and then averaged these across neurons. Error bars were computed as standard errors of mean across trials.

### Generalized Linear Modeling

#### Temporal basis functions

We used raised-cosine temporal basis functions to model the latencies between environmental events, firing rates, and kinematics. We used 4 basis functions with equal widths of 400 ms, and equispaced from each other with centers separated by 200 ms. We convolved each covariate time series with its respective basis set and then used these to predict firing rates. To prevent discontinuities between trial epochs, we zero-padded each trial with 500 ms (i.e., 50 time bins of 10 milliseconds, each), concatenated them, convolved the zero-padded time series, and then removed the zero-padding.

#### Model fitting

We fit models using the Matlab glmnet package which solves the convex maximum-likelihood optimization problem using coordinate descent (Hastie et al., 2009; Friedman et al., 2010). To prevent overfitting, we regularized model fits using elastic-net regularization [40]. We did not optimize the hyperparameters, but we found that a choice of *λ* = 0.1 (which determines the weight of the regularization term) and α = 0.1 (which weights the relative extent of L_1_ and L_2_ regularization) resulted in comparable training and test-set errors, and therefore did not inordinately over-fit or under-fit the data. We also cross-validated the model by fitting it to one random half of the trials and evaluating it on the other half. To evaluate model goodness of fit, we computed the pseudo-*R*^*2*^, which is related to the likelihood ratio. The idea of the pseudo-*R*^*2*^ metric is to map the likelihood ratio into a [0, 1] range, thus extending the idea of the linear *R*^*2*^ metric to non-Gaussian target variables. We used McFadden’s definition of pseudo-*R*^*2*^ [41, 23, 25]. For each neuron, we computed bootstrapped 95% confidence intervals of the pseudo-*R*^*2*^s.

#### Model comparison

To quantify whether individual covariates explain unique firing rate variance, we used partial models, leaving out the covariate of interest and comparing this partial model against the full model. To quantify this nested model comparison, we also used the relative pseudo-*R*^*2*^ metric. We obtained 95% confidence intervals on this metric using bootstrapping, for each cross-validation fold. We then treated the minimum of the lower bounds and the maximum of the upper bounds across cross-validation folds as confidence intervals. From these CIs, we approximated 2σ and 5σ significance levels, by calculating appropriate lower bounds for each significance level and comparing these lower bounds against zero.

## Supporting Information

S1 File Supporting Information file containing 5 supporting figures (A–E) and one supporting table (A). (DOCX)
