# Supplementary material for: Premotor and Motor Cortices Encode Reward
Source: PLoS One. 2016 Aug 26;11(8):e0160851. doi: 10.1371/journal.pone.0160851 (PMC5001708; doi:10.1371/journal.pone.0160851)
Supplement: S1 File — (DOCX) [file pone.0160851.s001.docx]

***Matching kinematics does not explain away reward signal***

Motor cortical activity strongly predicts the kinematics of movement, such as its velocity and direction [3, 4]. More confident or highly motivated reaches may be initiated earlier or performed faster, and may thus result in neural firing rate differences between successful and unsuccessful trials that are actually unrelated to reward. Two aspects of our task design could have potentially influenced variables such as confidence or motivation. First, even before movement onset, cue uncertainty can influence movement-planning confidence — the target location can be inferred with higher confidence from low-uncertainty cues than high-uncertainty cues. Second, after the trial completion, failure to secure reward might increase the urgency or desire to secure a reward in the next trial. Therefore, we asked whether kinematics — including latency of the return movement onset, instantaneous velocity, and acceleration — were systematically different between rewarded and unrewarded trials. Indeed, we found that monkeys started their return movement earlier for unrewarded trials, presumably because drinking the juice reward costs time and attention. The trial-averaged velocity and acceleration traces, when aligned to the time of reward (Fig. A(A), left panel), revealed small but significant differences between successful and unsuccessful trials. Therefore, any differences in neural firing rates related to reward might actually be attributed to kinematic differences. To control for these differences before analyzing the neural data, we matched the kinematics across conditions from 200 ms onwards after the reward (Fig. A(A), right panel) by selectively subsampling trials with similar return-movement peak velocities and latencies. For Monkey M, we included all trials where the peak velocity was between 11 and 16 cm/s, and the peak time was between 550 and 950 milliseconds. For Monkey T, we included all trials where the peak time was between 550 and 950 milliseconds, with no restriction on peak velocity.

**
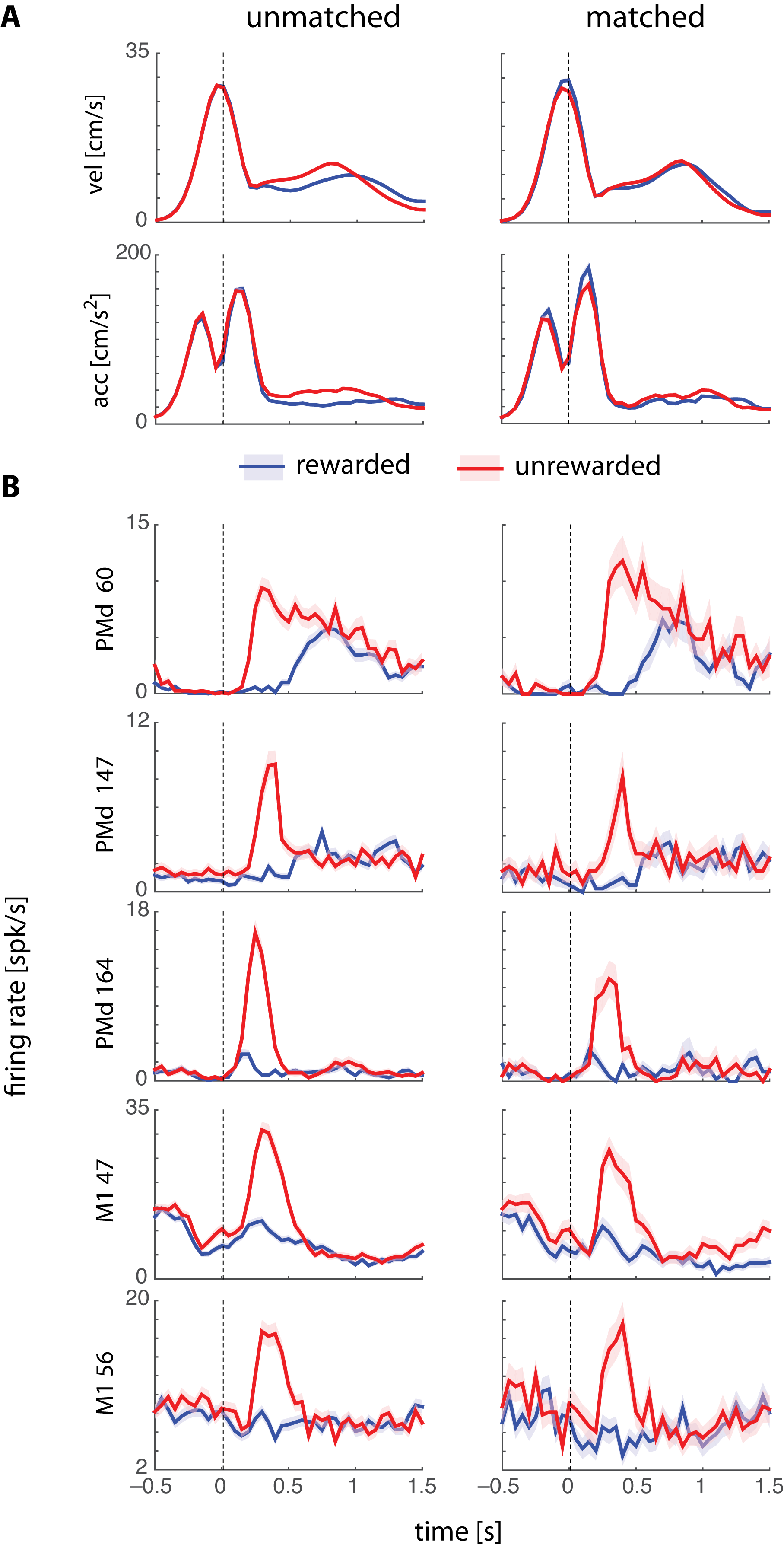
**

**Figure A.** (**A**) Velocity and acceleration traces for rewarded (blue) and unrewarded (red) conditions averaged across all trials (unmatched) and across trials selected to have similar latencies and amplitudes for one representative session (Monkey M, session 4). The dashed line at zero represents the reward onset. (**B**) PSTHs of example neurons (mean ± SEM) from the same representative session for unmatched and matched conditions.

**Table A.** **Number of rewarded and unrewarded trials before and after matching them for kinematics.**

| Monkey | Session | Before  n_rewarded | matching  n_unrewarded | After  n_rewarded | matching  n_unrewarded |
| --- | --- | --- | --- | --- | --- |
| M | 1 | 368 | 217 | 43 | 102 |
| M | 2 | 344 | 260 | 39 | 144 |
| M | 3 | 183 | 122 | 53 | 50 |
| M | 4 | 426 | 265 | 112 | 98 |
| M | 5 | 412 | 244 | 50 | 80 |
| M | 6 | 194 | 145 | 55 | 76 |
| T | 1 | 254 | 104 | 40 | 68 |
| T | 2 | 122 | 83 | 4 | 25 |
| T | 3 | 121 | 84 | 7 | 55 |

***Putative reward signal is not related to intrinsic success***

Because all successful trials were rewarded, it was not possible to tell whether the motor system actually encoded a failure in accurate movement completion, or the absence of extrinsic reward. Therefore, in a separate control experiment, we attempted to make this distinction by including a set of catch trials during the low uncertainty condition. In these catch trials the monkey was not rewarded, despite being successful. By comparing PSTHs for regular trials with PSTHs for these catch trials, we found that firing rates increased for any unrewarded trial, whether or not the animal had been successful (Fig. B), Therefore, PMd and M1 neurons encode the presence or absence of extrinsic reward, not intrinsic success or task outcome.

**
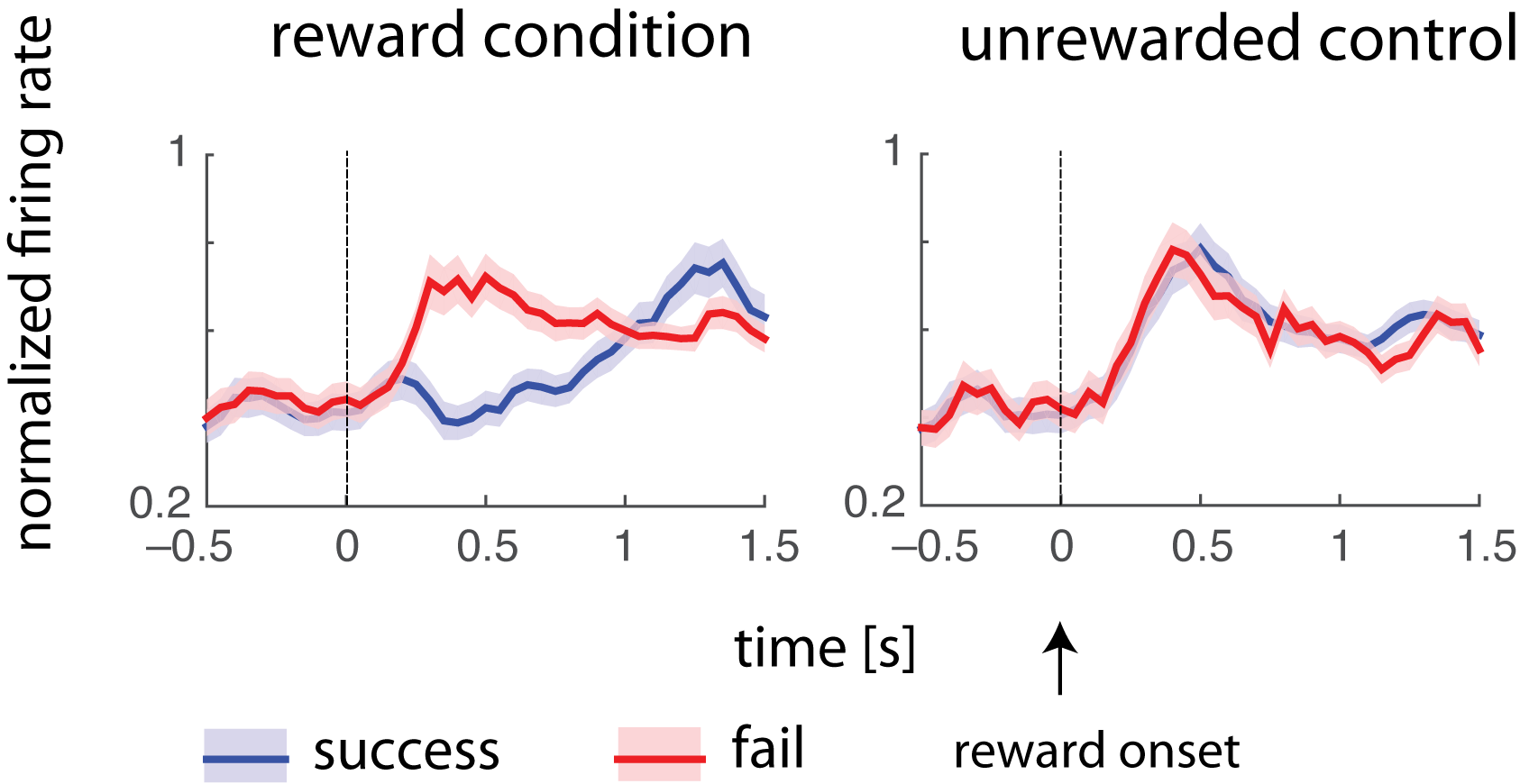
**

**Figure B.** Tuning to extrinsic reward, not intrinsic success. PMd neurons increased their firing rates (trial-averaged, normalized, mean ± SEM) when no reward was received, for successful as well as unsuccessful trials. The dashed line at zero indicates the reward onset time.

***Reward signal was not related to prediction error***

Dopaminergic neurons in the midbrain and prefrontal cortex are known to encode the mismatch between the magnitude of expected and obtained reward [5]. This difference is known as the reward prediction error, and is a useful learning signal in computational models of reinforcement learning [1, 2]. Therefore, we asked if the firing-rate increases in PMd and M1 resulting from the absence of reward might encode reward-prediction errors. Expected reward likelihood was indirectly related to the visual uncertainty of the target cue, which was displayed unpredictably in each trial by sampling from one of two distributions — a narrow one (low uncertainty) and a broad one (high uncertainty). Monkeys were more successful in low uncertainty trials and thus may have associated them with greater expectation of reward. A violation of this expectation would result in a reward-prediction error; a neural code for reward-prediction error magnitude would predict higher firing rates for successful high-uncertainty and failed low-uncertainty trials. To this end, we compared reward-aligned PSTHs for rewarded and unrewarded trials, separately for high and low uncertainty conditions. We found that unrewarded trials always had higher firing rates than did rewarded trials regardless of cue uncertainty (example neurons in Fig. C). Thus, unlike midbrain dopaminergic neurons, motor cortical neurons do not encode reward-prediction errors.


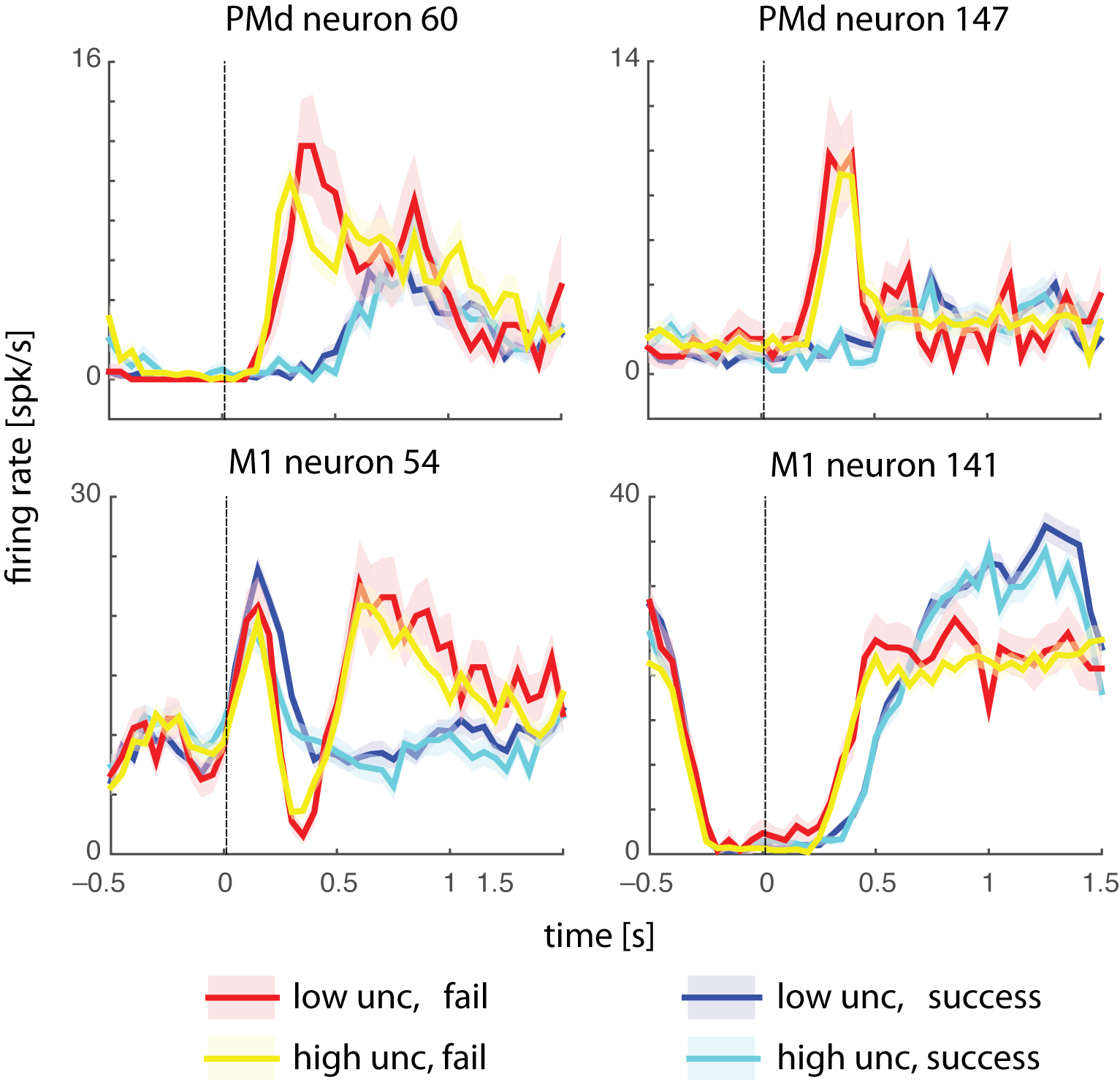


**Figure C.** No effect of uncertainty on reward encoding. PSTHs aligned to the time of reward (dashed line at zero) for example neurons from PMd and M1. The averages are separated by both trial outcome (rewarded or unrewarded) and cue uncertainty (low vs. high). Firing rates were strongly modulated by reward but not uncertainty, suggesting that the code is not related to reward-prediction error.

***Reward signal is distinct from return movement plan***

The post-reward-related increase in firing rates could potentially be explained by the fact that consuming the reward costs time and attention. Since consuming the reward takes attention away from planning the return movement, it could be that the return reach plan is better attended to in the unrewarded trials. If this were the case, the apparent reward-related activity that we observed should be explained by the return movement plan. One test for this confound is to examine whether the putative reward activity is spatially tuned to the return reach direction. We tested for this possibility by constructing a spatio-temporal activity plot over the entire population, in which activity was averaged across both neurons and trials, separated into bins by the difference between each neuron’s preferred direction and the return-reach direction of the trial (Fig. D). We used 64 uniformly spaced directional bin centers between –π and π, and time bins of 25 milliseconds. Although we observed a slight increase in the activity profile (Fig. D, top and middle panels: 200–500 ms after reward in PMd and 500–800 ms in M1) for return reaches toward the preferred direction, there was no significant difference between rewarded and unrewarded trials (Fig. D, bottom panels). These results suggest that the return reach plan is independent of the reward signal.


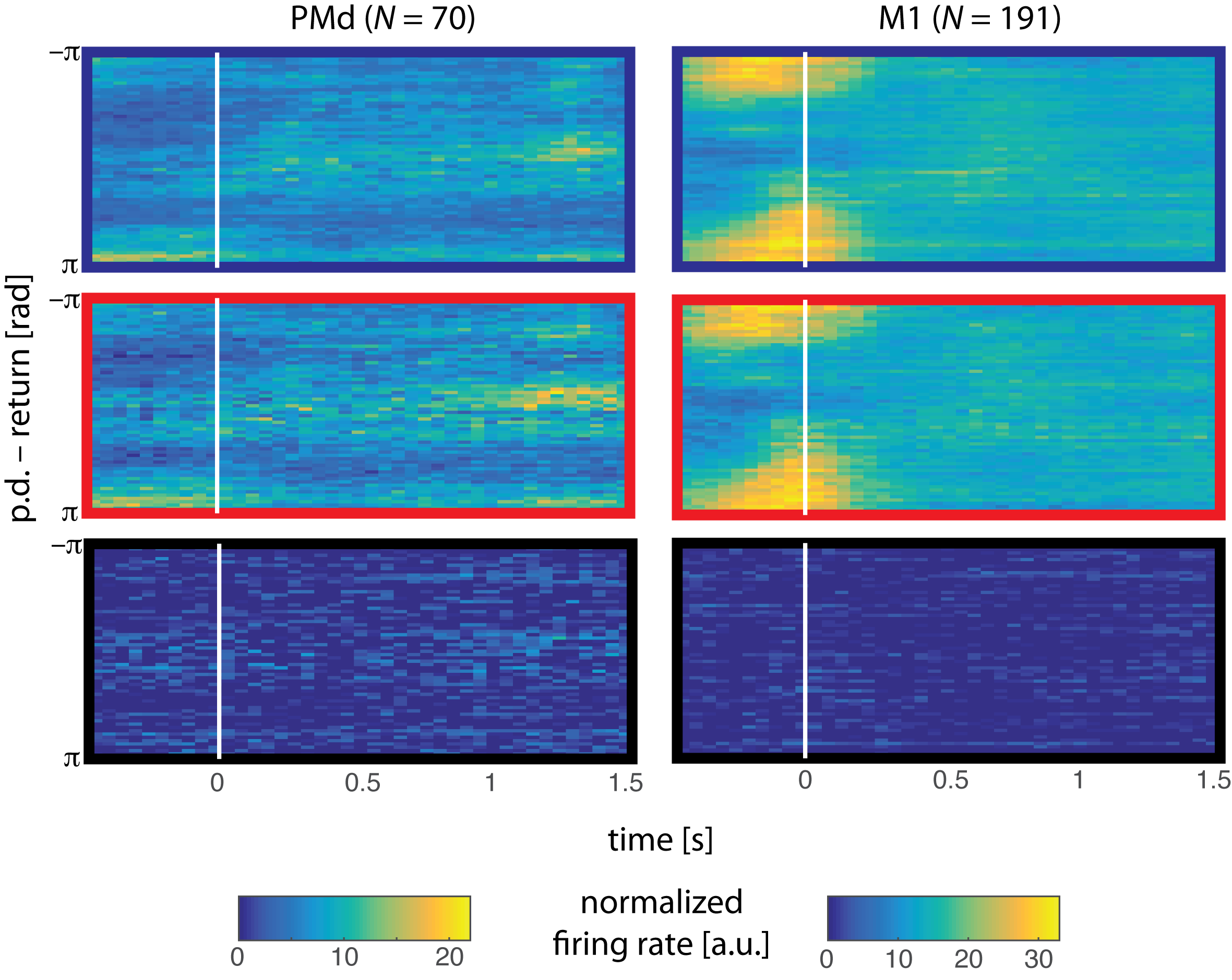


**Figure D.** Reward signal was not spatially tuned. Single session, monkey M: A spatiotemporal activity profile over the entire population, separated by the circular distance between the upcoming return reach direction and each neuron’s preferred direction, averaged across trials. Blue border: rewarded trials, Red border: unrewarded trials, Black border: difference. An increase in activity profiles for upcoming return reaches in a given neuron’s preferred direction for both classes of trials is indicative of the spatial tuning for the return reach plan. No significant differences were found between rewarded and unrewarded trials, suggesting that the spatially-tuned return reach plan was independent of reward encoding.

***Reward consumption control***

**
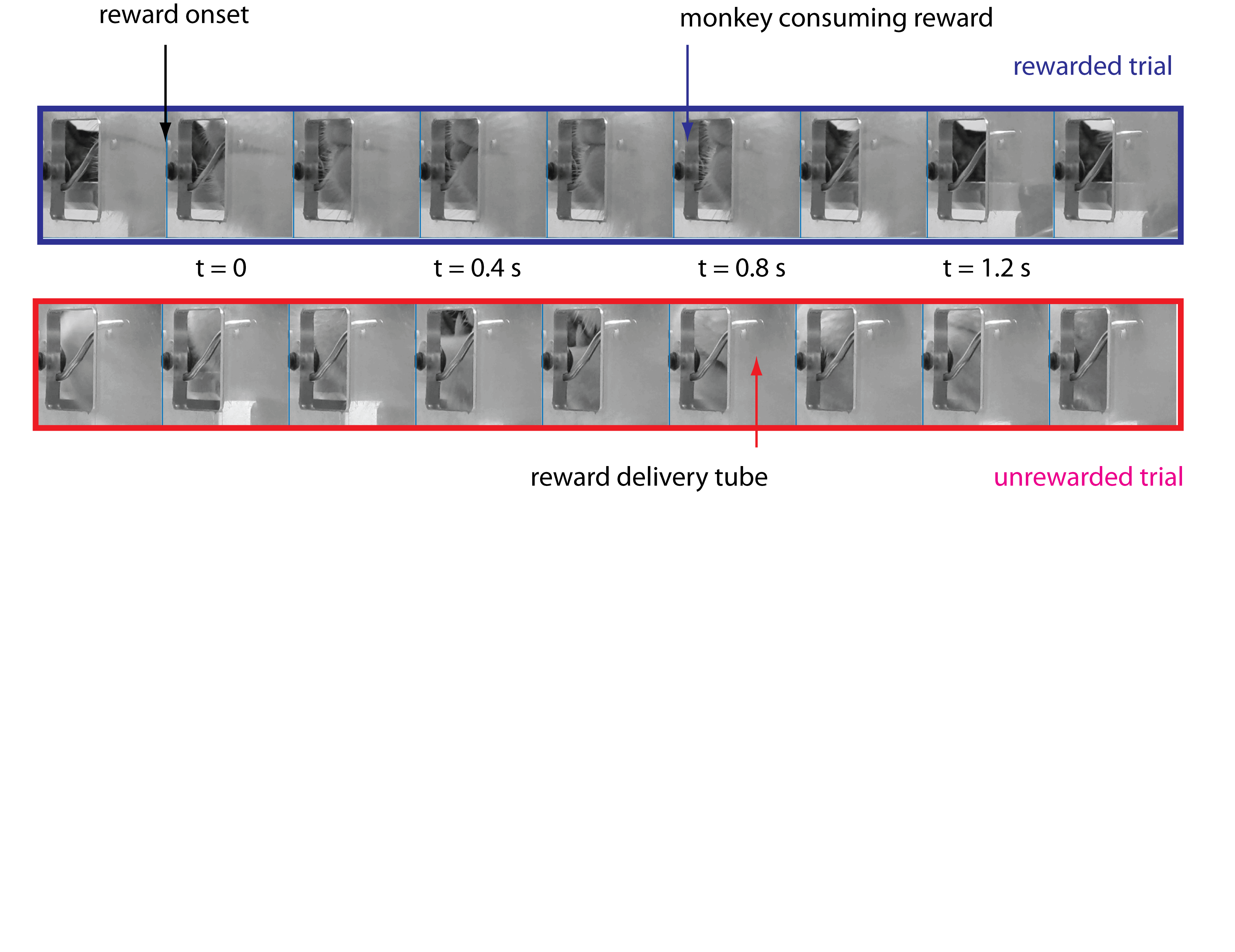
**

**Figure E.** **No mouth movements in unrewarded trials**. The increase in firing rate after lack of reward could potentially be explained by increased mouth or neck movement activity, e.g. if the monkey would suck harder at the tube. We filmed the monkey’s behavior after rewarded (example above) and unrewarded trials (example below) but found that in general, the monkey did not make contact with the tube after unrewarded trials.

**References**

[1] Hollerman JR, Schultz W. Dopamine neurons report an error in the temporal prediction of reward during learning. Nature neuroscience. 1998: 1, 304–309.

[2] O'Doherty JP, Dayan P, Friston K, Critchley H, Dolan RJ. Temporal difference models and reward-related learning in the human brain. Neuron. 2003: 38, 329–337.

[3] Georgopoulos AP, Kalaska JF, Caminiti R, Massey JT. On the relations between the direction of two-dimensional arm movements and cell discharge in primate motor cortex. The Journal of Neuroscience. 1982: 2, 1527–1537.

[4] Ashe J, Georgopoulos AP. Movement parameters and neural activity in motor cortex and area 5. Cerebral Cortex. 1994: 4, 590–600.

[5] Schultz W. Predictive reward signal of dopamine neurons. Journal of neurophysiology. 1998: 80, 1–27.
